# Comparison of bone mineral density and Fracture Risk Assessment Tool in Saudi women with and without type 2 diabetes mellitus: A cross-sectional study

**DOI:** 10.15537/smj.2022.43.7.20220144

**Published:** 2022-07

**Authors:** Eman M. Alfadhli, Ahad S. Alsharif, Razan A. Alharbi, Salma S. Alalawi, Shafigah E. Darandari, Showq A. Alsaedi, Shuruq O. Alharbi

**Affiliations:** *From the Department of Internal Medicine, College of Medicine, Taibah University, Al Madinah Al Munawarah, Kingdom of Saudi Arabia.*

**Keywords:** diabetes mellitus, type 2 diabetes mellitus, bone density, osteoporosis, FRAX

## Abstract

**Objectives:**

To compare the bone mineral density and the fracture risks in Saudi women with and without type 2 diabetes mellitus (T2DM).

**Methods:**

This cross-sectional study was carried out at Taibah Early Diagnostic Center, Al Madinah Al Munawarah, Saudi Arabia. A total of 465 women with and without T2DM aged ≥40 years who visited the center for a dual-energy X-ray absorptiometry scan between December 2020 and July 2021 were randomly selected. The 10-year probabilities of major osteoporotic fracture (MOF) and hip fracture (HF) were calculated using the Abu Dhabi Fracture Risk Assessment Tool (FRAX) with and without adjustment for T2DM. The adjustment was made by setting rheumatoid arthritis as the equivalent risk for T2DM in the FRAX. Bone mineral density values and the FRAX scores were compared between women with T2DM and non-diabetes.

**Results:**

Of 465 women, 214 had T2DM, and 251 were non-diabetics. The mean age of women was 59.42±7.9 years. There were no significant differences in mean age, menopausal status, height, weight, and body mass index between T2DM and non-diabetic women. Bone mineral density values and the unadjusted FRAX scores were comparable between the 2 groups. However, after adjusting FRAX for T2DM, the FRAX for MOF and HF became significantly higher in T2DM women (*p*=0.000 and *p*=0.004).

**Conclusion:**

In Saudi women with T2DM, unadjusted FRAX underestimated the risk of MOF and HF. Type 2 diabetes mellitus should be included as one of the clinical risk factors for fracture in future versions of the FRAX score.

**O**steoporosis and diabetes mellitus are 2 common chronic diseases that increase with aging.^1^ Osteoporosis is marked by a loss of bone mass as well as changes in bone microstructure, which leads to increased bone fragility and fracture risk.^2^ Diabetes is also linked to weakened bones and a higher risk of fracture.^3^

Osteoporosis is detected using a dual-energy X-ray absorptiometry (DXA) scan, which identifies bone mineral density (BMD) at the hip and lumbar spine and compares bone mass to a healthy young adult of the same gender (T-score). According to the World Health Organization (WHO), osteoporosis is defined as a T-score of ≤ -2.5, and T-scores between -1 and > -2.5 is considered osteopenia, and T-value of >-1 is normal BMD.^4^ Despite its potential to stratify fracture risk, BMD values from DXA have a low sensitivity. Many fractures happen in people who do not meet the BMD criterion for osteoporosis, indicating that bone strength and fracture risk are influenced by factors other than BMD.^5^ As a result, the fracture risk assessment (FRAX) score, a risk algorithm that incorporates several risk factors for fracture, was developed.^6^ The FRAX tool was developed to calculate the 10-year probability of major osteoporotic fracture (MOF) - at the hip, spine, forearm, and humerus - and the 10-year probability of hip fracture (HF) in both genders from the ages of 40-90 years. Listed risk factors for fracture in the current FRAX tool are; age, gender, body mass index (BMI), previous fragility fracture, hip fracture in parents, smoking, alcohol intake, secondary osteoporosis, rheumatoid arthritis, and glucocorticoid use, with or without adding the measured femoral neck BMD.^7^

Both types of diabetes, type 1 and type 2 diabetes mellitus (T1DM and T2DM), have been associated with increased fracture risk. Reduced bone mass and increased fracture risk has been reported in T1DM.^8^ Low BMD in T1DM patients could be caused by a failure to attain peak bone mass as well as low insulin levels, as insulin may play a role in bone growth.^9,10^ The situation in T2DM is controversial, with observations of raised, reduced, or maintained bone density displaying the heterogeneity of T2DM.^8^ Microarchitectural changes that decrease bone quality rather than bone quantity may represent the disparity between BMD and the risk of fracture in patients with T2DM.^11^ Type 1 diabetes mellitus is listed among the secondary causes of osteoporosis in the current formulation of the FRAX score; however, T2DM status is not. It was demonstrated that FRAX algorithm undervalues the risk of fractures in T2DM patients.^12^ Many techniques have been suggested to enhance FRAX’s implementation in T2DM patients, such as utilizing rheumatoid arthritis (RA) input in the FRAX, using the trabecular bone score (TBS)-adjustment, lowering the T-score of the femoral neck input to FRAX by 0.5 standard deviation, and raising the age input to FRAX by 10 years.^5^

The objective of the current study was to assess the BMD and the fracture risks in Saudi women with T2DM, with and without adjustment of FRAX, compared to non-diabetic women.

## Methods

This cross-sectional study was carried out at Taibah Early Diagnostic Center, Al Madinah Al Munawarah, Saudi Arabia, between December 2020 and July 2021. The center was chosen based on the availability and accessibility of the sampling subjects. Saudi women aged 40-90 years old (since FRAX is only utilized in this age range) who were referred to the center for a DXA scan with or without T2DM met the inclusion criteria. Non-Saudi women, T1DM, cancer, and those with RA were excluded as we used RA to adjust for T2DM in the FRAX model. A total of 504 women were randomly selected, and 465 were included in the study after applying the inclusion/exclusion criteria.

The study was approved by the Research Ethics Committee of Taibah University, the College of Medicine, and Taibah Early Diagnostic Center, Al Madinah Al Munawarah, Saudi Arabia, and carried out according to the principles of the Helsinki Declaration. All participants provided informed consent.

An English questionnaire designed by google forms was used to collect women’s data. Age, menopausal status, years since menopause, calcium and vitamin D supplements intake, and fracture risk factors that are involved in the FRAX model were recorded by the study team. These include prior fragility fracture (defined as a fracture that occurred at skeletal sites associated with osteoporosis such as hip, vertebrae, forearm, and humerus but was not caused by external trauma), parental HF, current smoking, drinking alcoholic, the use of glucocorticoids, and secondary causes of osteoporosis such as malabsorption, cushing syndrome, hyperparathyroidism, long-standing untreated hyperthyroidism, chronic organ failure, hypogonadism, or premature menopause. Tendency to fall and the causes for that, such as balance problems, dizziness, weak legs, limping or difficulty walking, and tiredness or fatigue, were also recorded. The presence or absence of T2DM categorized subjects into 2 groups: T2DM and non-diabetics. Diabetes was obtained by self-report. Weight (kg) and height (cm) were measured, and BMI was computed by dividing weight (kg) by height (m^2^) for all women.

Bone mineral density scans were carried out and analyzed using DXA Hologic (Hologic, Inc, Waltham, MA, USA). The lumbar spine (L1-L4) BMD, as well as bilateral proximal femurs (total hip and femoral neck), were measured by DXA scan. The WHO criteria were used to diagnose osteoporosis.^4^ A comparison was carried out between the 2 groups (T2DM and non-diabetics) regarding BMD results (g/cm^2^), T and Z scores of the femur neck, and the lumbar spine.

The 10-year probability of MOF and HF was calculated with femoral neck BMD utilizing the Abu Dhabi FRAX tool (Saudi FRAX was not available at the study time).^6,13^ The least femoral neck BMD (g/cm^2^) was used in the FRAX model. In the T2DM group, we calculated the FRAX one time with and one time without the adjustment of the predictive value of fracture risk by selecting RA input in the FRAX as a comparable variable for T2DM.^14^ Thus, for women with T2DM, calculations of FRAX ended up with 4 results: MOF and HF without adjustment and MOF and HF with adjustment. For the non-diabetic group, there were 2 results: MOF and HF. A comparison was made between the 2 groups regarding MOF and HF one time with and one time without adjustment for T2DM in the FRAX tool.

### Statistical analysis

Data were coded yes=1 and no=0, entered as numerical or categorical as appropriate, and analyzed using the Statistical Package for the Social Sciences, version 21.0 (IBM Corp., Armonk, NY, USA).

Means and SDs for continuous variables and frequencies and percentages for categorical variables were used to present demographic and clinical features of women with and without T2DM. We used Student’s t-tests for continuous variables and Chi-square tests for categorical variables to test for discrepancies in the baseline characteristics, BMD, and FRAX (MOF and HF) between women with and without T2DM. A *p*-value of <0.05 was considered significant.

## Results

Of the 465 women who were included; 214 had T2DM, and 251 were non-diabetics. The mean age of the women was 59.4±7.9 years (range: 40-83); with 77.6% of them being postmenopausal. There were no significant differences between the 2 groups in the baseline characteristics such as age, age groups, menopausal status, height, weight, BMI, calcium, or vitamin D intake. Table 1 shows the baseline characteristics of all women and the differences between the 2 groups.

**Table 1:** - Demographic data and the baseline characteristics of 465 women aged ≥40 years (40-83); and the differences between type 2 diabetes mellitus and non-diabetics groups.

Variables	All (N=465)	Non-DM (n=251)	T2DM (n=214)	*P*-value
*Age (years)*	59.4±7.9	59.3±7.9	59.6±8	0.723
40-49 years	46 (9.9)	26 (10.4)	20 (9.3)	
50-59 years	204 (43.9)	115 (45.8)	89 (41.6)	
60-69 years	149 (32.0)	77 (30.7)	72 (33.6)	
70 years and above	66 (14.2)	33 (13.1)	33 (15.4)	
Postmenopausal	361 (77.6)	193 (76.9)	168 (78.5)	0.738
Height (cm)	154.0±6.9	154.4±5.7	153.5±8	0.167
Weight (Kg)	75.3±16.3	75.4±15.5	75.1±17.1	0.860
BMI (kg/cm^2^)	31.5±6.3	31.5±6.1	31.4±6.5	0.976
On calcium supplements	244 (52.5)	132 (52.6)	112 (52.3)	1.000
On Vitamin D supplements	286 (61.5)	153 (61)	133 (62.1)	0.848

Data are presented as numbers and precentage (%), mean ± standard deviation (SD). DM: diabetes mellitus, T2DM: type 2 diabetes mellitus, BMI: body mass index

There were no significant differences in the DXA scan parameters; BMD, T-score, and Z-score between T2DM and non-diabetics. In all women, the prevalence of osteopenia was 50.1 and the prevalence of osteoporosis was 34.0. Although osteopenia was more in non-diabetics than in T2DM and osteoporosis was more in T2DM, the results did not reach statistical significance (Table 2). There was no significant difference between the 2 groups in the rate of secondary osteoporosis, nor fracture risk factors (Table 3). The rate of prior fragility fracture was 13.1%, with no significant differences between the 2 groups. Tendency to fall was the most typical reason for fragility fracture, affecting more than 50% of the studied women (Table 4).

**Table 2:** - Measured values of the dual-energy X-ray absorptiometry scan and the prevalence of osteopenia and osteoporosis in 465 women aged ≥40 years and the differences between the type 2 diabetes mellitus and non-diabetics groups.

Variables	All (N=465)	Non-DM (n=251)	T2DM (n=214)	*P*-value
R FN BMD (g/cm^2^)	0.7±0.1	0.7±0.1	0.7±0.1	0.100
R FN T score	-1±1.1	-0.9±1.2	-1.1±1.1	0.096
R FN Z score	0.2±1.0	0.2±1	0.12±1	0.252
L FN BMD (g/cm^2^)	0.7±0.1	0.7±0.1	0.7±0.1	0.193
L FN T score	-1±1.2	-0.9±1.3	-1.1±1.1	0.187
L FN Z score	0.1±1.2	0.2±1.2	0.07±1.3	0.188
Lumbar spine BMD (g/cm^2^)	0.8±0.1	0.8±0.1	0.8±0.1	0.664
Lumbar spine T score	-1.8±1.2	-1.8*±*1.3	-1.8*±*1.2	0.592
Lumbar spine Z score	-0.4±1.2	-0.4±1.2	-0.4±1.2	0.660
Osteopenia, n(%)	233 (50.1)	133 (53)	100 (46.7)	0.193
Osteoporosis, n (%)	158 (34.0)	78 (31.1)	80 (37.4)	0.169

Data are presented as mean ± standard deviation (SD). DM: diabetes mellitus, T2DM: type 2 diabetes mellitus, R: right, L: left, FN: femur neck, BMD: bone mineral density

**Table 3:** - Prevalence of fracture risk factors of 465 women aged ≥40 years and the differences between the type 2 diabetes mellitus and non-diabetics groups.

Variables	All (N=465)	Non-DM (n=251)	T2DM (n=214)	*P*-value
Secondary osteoporosis	22 (4.0)	10 (4.0)	12 (5.6)	0.512
On glucocorticoids	44 (9.5)	25 (10)	19 (8.9)	0.752
Malabsorption	12 (2.6)	4 (1.6)	8 (3.7)	0.240
Cushing syndrome	0 (0.0)	0 (0.0)	0 (0.0)	NA
Hyperparathyroidism	3 (0.6)	3 (1.2)	0 (0.0)	0.253
Thyrotoxicosis	7 (1.5)	3 (1.2)	4 (1.9)	0.708
Liver failure	0 (0.0)	0 (0.0)	0 (0.0)	NA
Current smoking	7 (1.5)	6 (2.4)	1 (0.5)	0.130
Parent fractured hip	27 (5.8)	12 (4.8)	15 (7)	0.326
On osteoporosis medications	90 (19.4)	48 (19.1)	42 (19.6)	0.907

Data are presented as numbers and precentage (%). DM: diabetes mellitus, T2DM: type 2 diabetes mellitus, NA: not applicable

**Table 4:** - The rate of prior fragility fracture and associated risks of 465 women aged ≥40 years and the differences between the T2DM and non-diabetics groups.

Variables	All (N=465)	Non-DM (n=251)	T2DM (n=214)	*P*-value
Low trauma fracture	61 (13.1)	32 (12.7)	29 (13.6)	0.891
Hip fracture	26 (5.6)	12 (4.7)	14 (6.5)	0.445
Spine fracture	8 (1.7)	6 (2.4)	2 (0.9)	0.260
Arm fracture	29 (6.2)	14 (5.6)	15 (7.0)	0.612
Tendency to fall	252 (54.2)	132 (52.6)	120 (56.1)	0.457
Balance problem	32 (12.7)	13 (9.8)	19 (15.8)	0.186
Limping or difficulty walking	64 (25.4)	40 (30.3)	24 (20)	0.082
Weak legs	84 (33.3)	46 (34.8)	38 (31.7)	0.688
Feeling dizzy	69 (27.4)	34 (25.8)	35 (29.2)	0.574
Feeling tired and fatigue	89 (35.3)	44 (33.3)	45 (37.5)	0.512

Data are presented as numbers and precentage (%). DM: diabetes mellitus, T2DM: type 2 diabetes mellitus

**Figure 1:**
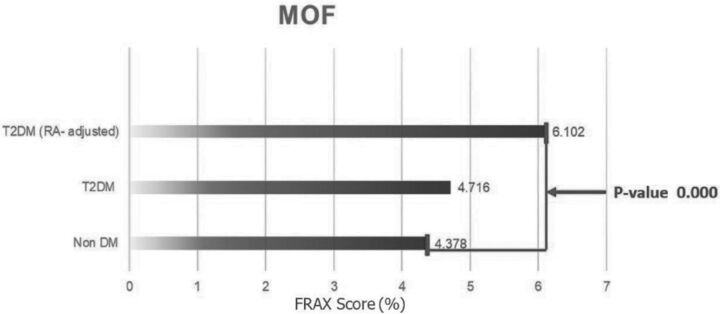
- The fracture risk assessment (FRAX) score for major osteoporotic fracture in type 2 diabetes (T2DM) women versus non-diabetes with and without rheumatoid arthritis (RA) adjustment. MOF: major osteoporotic fracture, non-DM: non-diabetes

**Figure 2:**
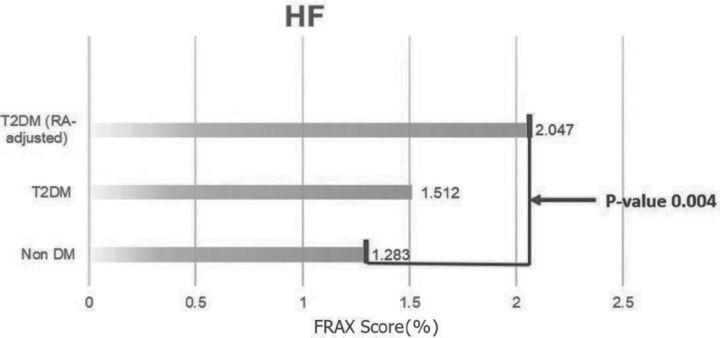
- The fracture risk assessment (FRAX) for hip fracture (HF) in type 2 diabetes (T2DM) women versus non-diabetes with and without rheumatoid arthritis (RA) adjustment. non-DM: non-diabetes

There were no significant differences in the FRAX score between T2DM and non-diabetic groups. However, when FRAX risk was adjusted for T2DM, the FRAX scores for both MOF and HF became significantly higher in T2DM women than non-diabetics. A *p*-value was 0.000 for MOF (Figure 1) and 0.004 for HF (Figure 2).

## Discussion

Diabetes mellitus is one of the most prevalent chronic disorders, affecting most of the body tissues and causing significant morbidity and mortality. Diabetes can cause bone loss, which can result in osteopenia, osteoporosis, and fragility fractures.^1,15^ According to several reports, low bone mass in T1DM patients at the hip, femoral neck, and spine may increase the risk of bone fracture. In T2DM, on the other hand, data are contradictory, and the specific mechanism of increased fragility fractures is uncertain.^16^ In the present study, Saudi women with T2DM had an equivalent BMD measured at the femoral and lumbar spine to non-diabetic women, thus converging with some data.^17-19^ A study by Zhou et al^17^ that included 890 age-matched women after menopause with T2DM and 689 non-diabetic women and divided the participants into obese (BMI of ≥25 kg/m^2^) and non-obese (BMI of <25 kg/m^2^) showed no significant differences in BMDs, T-scores, or Z-scores at the total hip and femoral neck in diabetic and non-diabetic women with BMI of ≥25 kg/m^2^, while the Z-score at the lumbar spine was higher in T2DM women. After adjusting for BMI, other study reported no difference in BMD between T2DM and control subjects at any site.^18^ Similarly, a study carried out by Bonaccorsi et al^19^ reported no significant difference in the BMD values between postmenopausal women with T2DM and control subjects; however, TBS was found to be considerably lower in T2DM women. In contrast to our results, other literature found that patients with T2DM have greater BMD values than non-diabetic patients.^16,20-24^ A study carried out by Bayani et al^16^ demonstrated that BMD of the lumbar spine was significantly greater in older women with diabetes than non-diabetes, however, no difference was observed for femoral neck BMD between the 2 groups. In the latter study, patients with diabetes had a lower prevalence of osteoporosis and osteopenia than non-diabetics.^16^ Conversely, Kao et al^20^ reported greater BMD at the hip but not at the spine or forearm in Mexican-American women with T2DM compared to non-diabetic counterparts. A study carried out by Oei et al^21^ found that the BMD at the femoral neck and lumbar spine was greater in diabetic subjects than in non-diabetic subjects. A Japanese study which enrolled both genders reported that T2DM patients had a higher BMD value than controls.^22^ Similarly, a study in postmenopausal Asian women found that the non-diabetic group had a lower BMD than the T2DM group.^23^ Likewise, in another retrospective clinical study from Rome, T2DM patients were found to have significantly higher mean femoral neck BMD and T-score values than non-diabetic.^24^ The reason for the incongruity in the BMD results for T2DM versus non-diabetic subjects in various studies is not clear; however, differences in the age, BMI, menopausal status, and other baseline characteristics between T2DM subjects and the non-diabetics in some studies may partly clarify this inconsistency. Type 2 diabetes mellitus women in the current study had comparable values of age, height, weight, BMI, calcium and vitamin D supplements intake, and menopausal status to the non-T2DM group. This uniformity may clarify why our T2DM women had comparable BMD to non-diabetics.

A FRAX tool calculates the 10-year odds of major osteoporotic fracture and hip fracture in individuals of both genders over 40 years. It combines multiple clinical risk factors with or without femoral neck BMD to improve fracture risk assessment. In the present study, we calculated the FRAX tool with the femoral neck BMD for both groups and with and without RA-adjustment for T2DM women. Among women with T2DM, unadjusted FRAX risk was not different from non-diabetics, thus concurring with the findings of some papers and different from others. A study carried out by Ohira et al^25^ included that 47,389 Japanese women showed FRAX for major osteoporotic fracture risk was not significantly different between individuals with T2DM and non-diabetics, despite the fracture rate in the past 5 years being significantly higher in the diabetic group. In contrast, a study carried out by Wang et al^23^ that included 1014 individuals (500 T2DM) found that T2DM had a higher FRAX compared with the non-diabetics group, even after controlling age, gender, BMI, smoking condition, alcohol intake, and low-density lipoprotein levels. Conversely, in another study, the FRAX of both MOF and HF was considerably lower in the entire sample and in males with diabetes than in control subjects, but no significant differences were seen between women with diabetes and control subjects.^26^ Patients with diabetes, on the other hand, had a considerably higher rate of prior fractures than control subjects in the entire cohort.^26^ Some studies indicated that the FRAX score’s mean values were lower in patients with T2DM than in patients without diabetes; however, after controlling T2DM in the FRAX model, the differences between the 2 groups were negated.^24,27^ In the current study, the FRAX score became significantly higher in the T2DM women than in non-diabetics after the adjustment of T2DM in the FRAX. As demonstrated in several studies, the FRAX algorithm misjudges the risk of fractures in T2DM patients, and this is due to the fact that T2DM is not incorporated in the current FRAX algorithm.^28^ Many approaches to improve FRAX performance in T2DM patients have been suggested; one of those methods was applying RA input to the FRAX tool for patients with T2DM.^25,29^

In the present study, there were no significant differences in rate of prior fragility fracture between the 2 groups. This finding is incongruent with other reports, which demonstrated an increase in the fracture risk in T2DM patients.^22,25,30^ The reason for this disparity may be linked to the relatively small sample size of our study and the dependence on self-report of previous fractures, which may be affected by recall bias and missing asymptomatic vertebral fractures. According to Bonaccorsi et al,^19^ previous fractures were approximately 3 times more common in diabetic women than in controls (13.8% versus 3.4%; *p*=0.02). Ohira et al^25^ demonstrated that the fracture rate in Japanese women with T2DM was considerably greater than in control subjects in the previous 5 years. Yamamoto et al^22^ noted an elevated risk of vertebral fractures in T2DM patients regardless of BMD or diabetes complications, implying that bone fragility in T2DM is determined by bone quality. Data from 3 prospective observational studies carried out in the United States of America found that adults with diabetes experienced a higher risk of fracture than those without diabetes.^30^ A prospective multicentral investigation is needed to compare the BMD and FRAX and the actual incidence of fractures during a long follow-up period in a larger population of Saudi women with and without T2DM.

The mechanisms underlying the link between diabetes and bone health remain unknown. Bone remodeling may be impaired in T2DM, and there may be alterations in the differentiation and function of the osteoblastic activity.^18^ Insulin may play an important function in maintaining normal bone formation by stimulating osteoblasts and indirectly decreasing collagen degradation.^31^ Type 2 diabetes mellitus individuals usually have an excess of insulin at the start of the disease, which may explain the increased bone mass in the early years of diabetes.^20^ Leslie et al^32^ found that diabetic patients have a biphasic risk of fracture, with a lower risk of osteoporotic fracture in newly diagnosed patients while higher fracture risks in patients with long-standing diabetes. Poor glycemic control was demonstrated to increase the risk of fracture in diabetic patients.^21^ Participants with poorly controlled diabetes had a 47-62% higher fracture risk than those without diabetes, according to a Rotterdam study.^21^ In contrast, those with well-controlled diabetes had the same risk as those who did not have diabetes.^21^ Chronic diabetes complications including diabetic neuropathy, retinopathy, and peripheral vascular disease all enhance the risk of fracture in diabetic patients.^33^ Long-term thiazolidinedione treatment raises the incidence of fractures in people with T2DM.^33^ In addition, hypoglycemic events increase the risk for falling and hence increase the fracture risk.^34^

### Study strengths and limitations

The first limitation is that we only collected data from one center, therefore we were unable to examine a larger sample size. Second, diabetes was determined by self-report, which may have led to the inclusion of women with diabetes among non-diabetic women. Finally, the current study’s cross-sectional methodology allows for the identification of associations rather than causative links between diabetes, BMD measurements, and FRAX scores. However, this study is necessary as it highlights the BMD and FRAX in Saudi women with and without T2DM. Another aspect of this research is that we calculated the FRAX score using BMD, which enhances the FRAX’s precision. Furthermore, T2DM and non-diabetes groups in our study were matched in all aspects of the risk factors of fracture, such as age, menopausal status, height, weight, BMI, and calcium and vitamin D supplements intake.

In conclusion, BMD values and the unadjusted FRAX scores are not different between Saudi women with T2DM and non-diabetes. However, the adjusted FRAX scores for both MOF and HF are significantly higher in the T2DM group. Unadjusted FRAX underestimated the fracture risks in T2DM Saudi women. Type 2 diabetes mellitus should be included as one of the clinical risk factors for fracture in future versions of the FRAX score.
